# Healthy Maine Partnerships: Policy and Environmental Changes

**Published:** 2009-03-15

**Authors:** Sarah Levin Martin, Dorean Maines, Debra Wigand, Maurice W. Martin, Pamela Bruno MacDonald, Marco Andrade, Ruth Dufresne, Laura Ronan, Michele Polacsek

**Affiliations:** Maine Center for Public Health; Maine Center for Disease Control and Prevention, Augusta, Maine; Maine Center for Disease Control and Prevention, Augusta, Maine; University of Maine at Farmington, and the Maine Center for Public Health, Farmington, Maine; Maine Center for Public Health, Augusta, Maine; Maine Center for Public Health, Augusta, Maine; Maine Center for Public Health, Augusta, Maine; Maine Center for Public Health, Augusta, Maine; Maine Harvard Prevention Research Center, Augusta, Maine

## Abstract

**Background:**

Tobacco settlement funds were used to establish the Healthy Maine Partnerships (HMPs) to reduce tobacco use, increase physical activity, and improve nutrition through local policy and environmental change.

**Context:**

The HMP model is a progressive approach to public health. It provides for coordinated efforts between state and local partners for health promotion and disease prevention. Community coalitions, supported with funding and guidance by the state, are the basis for policy and environmental change.

**Methods:**

The state awarded contracts and provided program guidance to foster policy and environmental change at the local level. The partnerships' efforts were assessed with a retrospective evaluation that consisted of 2 data collection periods conducted using the same tool. A survey booklet containing lists of possible environmental and policy changes was developed and mailed — once in 2005 and once in 2006 — to all 31 local partnership directors and school health coordinators who completed it. Additional data were collected from the local partnerships in the form of narrative reports required by their funder (Maine Center for Disease Control and Prevention).

**Consequences:**

All local partnerships implemented policy or environmental interventions to address tobacco use, physical activity, and nutrition during the period covered by the surveys (July 2002-June 2005 [fiscal years 2003-2005]). Cumulatively, more than 4,600 policy or environmental changes were reported; tobacco use policies represent most changes implemented. A second round of HMP funding has since been secured.

**Interpretation:**

Although the survey methodology had limitations, results suggest that much work has been accomplished by the local partnerships. Plans are to share success stories among partnerships, provide training, and continue to improve the public health infrastructure in Maine.

## Background

Heart disease is the leading cause of death in Maine, and stroke is the third leading cause (1) According to 2001 data, Maine's death rates for heart disease, cancer, and stroke were slightly higher than the national rate (1). Tobacco use, lack of physical activity, and poor nutrition are major contributors to these and other chronic conditions. According to 2001 data from the Behavioral Risk Factor Surveillance System (2) and the Youth Risk Behavioral Surveillance System (3), most Maine estimates were similar to national estimates. However, the rate of students who smoked more than 10 cigarettes per day on days they smoked was higher in Maine than the national rate (21.1% vs 14.4%) (3).

The Healthy Maine Partnerships (HMPs) were established by the Maine Center for Disease Control and Prevention (Maine CDC) in January 2001 as local intervention sites to decrease tobacco use and tobacco-related chronic diseases in Maine and improve the health of its residents. Initially, several Maine CDC programs supported local HMPs: Partnership For A Tobacco-Free Maine, Maine Physical Activity and Nutrition Program (PANP), Maine Cardiovascular Health Program (MCVHP), Community Health Promotion Program; and the Coordinated School Health Program (CSHP) jointly managed by the Maine CDC and Maine Department of Education. At that time, the MCVHP focused on primary prevention.

By using funds from the Master Settlement Agreement and with support from the Maine CDC, local HMPs focused on changing local policies and environments through community mobilization to reduce tobacco use, increase physical activity, and improve nutrition. We summarize the progress made by HMPs in their first few years (July 2002-June 2005).

## Context

Policy and environmental approaches influence physical activity and nutrition behaviors (4-6). Many of these strategies are also recommended as part of a comprehensive tobacco use control program where, for example, policies affecting environments (eg, smoking bans) are considered to be a best practice (7). Such changes support people who adopt healthier behaviors by providing community access to healthy options and environments, information, and incentives. Policy interventions focus on changing behaviors through regulations or laws, such as prohibiting smoking on worksite or school grounds. Environmental changes may be accomplished by using strategies such as building walking trails or promoting farmers' markets and community gardens.

Maine has a population of 1.3 million and a land area exceeding 30,000 square miles, which approximates that of the 5 other New England states combined (8). With a population per square mile of 41.3, Maine is the second most rural state in the United States (59.5% of the Maine population is defined as rural, compared with 21% nationally and 19.4% in New England) (8,9).

In a state so large and rural, the need for an extensive public health infrastructure is warranted. Before 2001, no statewide public health infrastructure existed in Maine beyond the state office and 2 city health departments in the 2 largest cities, Portland and Bangor, which are separated by 133 miles. Before Master Settlement Agreement monies were allocated, stakeholders met to begin planning for a public health system that would work in the state to reduce the burden of chronic disease, tobacco-related and otherwise. Community-based coalitions were chosen as the model. In 2000, a Governors' Summit, attended by former Surgeon General David Satcher, was convened to highlight the need for prevention and to promote the community-based coalition approach. Having a plan that was derived from stakeholder input and support was a key factor in securing Master Settlement Agreement funds for health care and disease prevention.

The community-based coalitions linked schools, health care systems, consumers, and community-based organizations to coordinate efforts to reduce barriers to practicing healthy behaviors. These coalitions shifted the focus of health from patient-focused to population-focused and were coordinated with, and empowered by, resources from the state to execute the HMP model.

Each local partnership had a lead agency operating as the fiscal agent and at least 1 collaborating school administrative unit (SAU). Most (n = 25) lead agencies for the partnerships were hospitals, 5 were nonprofit organizations, and 1 was a local university. Staffing of the partnerships included a local director hired by the lead agency and at least 1 school health coordinator (SHC) for each SAU. Approximately 50 full-time and part-time SHCs were hired to work within the 31 HMPs. Most HMPs (n = 25) also had a full-time or part-time youth advocacy coordinator, and 14 HMPs had additional staff (Appendix). Each of the 31 local HMPs was funded to reach specific service areas, which together cover most of the towns and organized territories in Maine.

## Methods

Participating HMPs were recruited by a formal request for proposals. Contracts were awarded on the basis of whether programs covered most of the state population and on the size of the population covered, among other considerations. Once contracts were awarded, the Maine CDC provided guidance by specifying requirements to change policies and environments, with a long-range goal of changing social norms. For example, "Action Packets" — 4 packets designed by the MCVHP and PANP — were provided to local HMPs to assist them with their efforts to increase participation in physical activity and improve nutrition behaviors.

In 2001, to commemorate the founding of local HMPs, the state sponsored planning, networking, and training meetings for all HMP grantees. Quarterly regional meetings were also held to allow for more networking and idea sharing. The meetings were an opportunity for the state to ensure it was providing adequate technical assistance and meeting evaluation needs of local HMPs. Guidelines and recommendations were developed and distributed, along with other resources, at these meetings and through other channels to educate participants. For instance, guidelines for the incorporation of a youth advocacy approach were developed and disseminated, as were recommendations, funding, and training for youth advocacy mentors and youth leaders. Project officers assigned to each local partnership provided ongoing technical assistance and conducted occasional site visits, regular conference calls, and other communications. To monitor the progress and impact of interventions that had been implemented, contractors evaluated efforts.

Quantitative findings were derived from 2 distinct administrations of the "Outcome Survey to Measure Statewide Progress: Local Healthy Maine Partnerships Policy and Environmental Changes" (Outcome Survey). The Outcome Survey was a paper-based, booklet-style survey that assessed policies and environmental changes newly in place and was administered by a contractor of the Maine CDC. The first administration covered July 2002 through June 2004 [fiscal years (FY) 2003 and 2004] and the second one covered July 2004 through June 2005 (FY 2005). The HMPs functioned throughout both administrations and continue to do so. The HMPs received announcement letters and e-mails before the Outcome Survey was mailed, followed by a series of phone calls to introduce the survey, remind recipients of the pending return date (April 2005 for the first administration and February 2006 for the second), and follow up on surveys not yet received. For HMPs in which there had been substantial staff turnover, the contractors made arrangements for former staff to complete the survey. To ensure that the staffing skills required to complete the survey were present, each HMP was asked to work as a team, with their community and school partners, to fill out a single booklet.

The survey covered 6 domains: 1) local partnership profile, 2) tobacco policy and environmental changes, 3) physical activity policy and environmental changes, 4) nutrition policy and environmental changes, 5) cardiovascular health prevention activities, and 6) activities within the Youth Advocacy Program (a program to train and empower youth to advocate for policy and environmental change). We focus on the policy and environmental changes regarding tobacco, physical activity, and nutrition. The settings assessed in the Outcome Survey included schools, worksites, hospitals, municipalities, colleges, and restaurants.

Qualitative findings were derived from the "Five-Year Review," which was conducted in 2006 and included narrative space to report process and progress. These reflections from local HMPs were captured in narrative format in 2 binders — 1 for the HMPs as a whole and 1 for the CSHP. The HMP binder included narrative sections about the partnership as a whole, the collaborations, the Youth Advocacy Program, and the CSHP (CSHP data were copied for the CSHP binder for the Maine Department of Education). Taken together, the quantitative and qualitative findings describe the experiences of local HMPs.

## Consequences

Most HMPs addressed tobacco, physical activity, and nutrition in several different settings (Table 1). The primary setting for tobacco efforts was municipalities, followed closely by schools and worksites, whereas the primary setting for physical activity and nutrition efforts was schools (Table 1).

The Figure describes the total number of policy changes accomplished by setting and focus area. Cumulatively, more than 4,600 policy or environmental changes were accomplished (2,418 for tobacco, 1,683 for physical activity, and 591 for nutrition).

**Figure.**
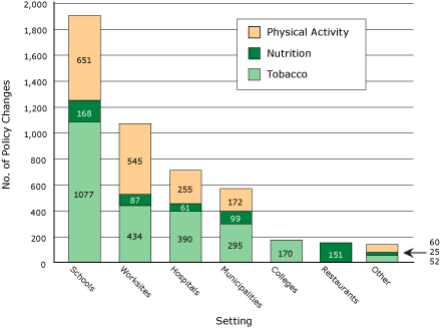
Cumulative policy and environmental changes accomplished, by setting and focus area, Healthy Maine Partnerships, fiscal years 2003-2005.

**Table:** 

Examples of changes made by local HMPs are provided below for each of the 3 focus areas. These examples are drawn from notes in the "Five-Year Review" and provide a closer look at a few of the successes of the community coalitions. Table 2 contains the most frequently reported change by behavior and setting on the basis of results from the Outcome Survey.

### Tobacco

- The greater Lewiston-Auburn area pioneered an effort to enforce clean air in multi-unit rental housing. Members of Healthy Androscoggin, responding to several citizens who complained about exposure to secondhand smoke, began research and initiated a survey to assess tenant and landlord views on this issue. On the basis of survey results, the Auburn Housing Authority established the first smoke-free housing policy for all of its units, the first in Maine and the third in the nation to do so.
- The Somerset Heart Health coalition reached out to businesses and encouraged the development of wellness councils as a key facet for the local chambers of commerce. Larger employers, such as Madison Paper Industries and New Balance Athletic Shoe, Inc, worked with the local HMP to implement a national program called "Quit and Win." Between 2004 and 2005, the program helped 62 people end their addiction to tobacco.
- Students at Hichborn Middle School, assisted by their local HMP, "Sprint for Life," wrote to the Howland town manager and to each member of the board of selectmen, requesting that Memorial Park, used by local youth for baseball and softball, be declared a smoke-free park with appropriate signage. The selectmen were so impressed by the youth and their commitment that they agreed to the request through a unanimous vote.

### Physical activity

- In the Calais area, School Union 106 developed a "lifelong physical activity program" by acquiring and storing kayaks, snowshoes, and cross-county skis donated through the work of volunteers and community groups. The sports equipment can be signed out free of charge by students, staff, and community members on weeknights and on weekends. In addition, an after-school program to promote physical activity and nutrition has been created, in which more than 60% of the students participate.
- The "Choose to be Healthy" coalition focused some of its community-based efforts on increasing the availability and use of walking and biking trails in the municipalities of York and Kittery.

### Nutrition

- In 2004, 2 HMPs, "Healthy Peninsula" and "Healthy Acadia," working with the cooperative extension service and county food pantry directors, realized that food stamp clients wanted access to affordable fruits and vegetables. Working together to overcome barriers, the effort to include local food options in food security efforts succeeded. The 2006 growing season resulted in more than 650 pounds of fresh produce donated to the local food pantries for distribution.
- In School Administrative District number 22, a collaborative process initiated by the local HMP (Bangor Health and Welfare) successfully changed the policy for vending machines. As a result, soda and unhealthy snacks have been permanently removed and replaced with healthy beverages and snacks. Seven schools made the change through the work of the HMP-funded SHC, the school staff, and the students (Table 2).

Progress was shared with the community in many ways. Newspaper articles and newsletters were the most commonly reported mode of dissemination. Results of the Outcome Survey have been formatted in newsletter fashion and shared with local HMPs and state legislators in an effort to further support for the partnerships.

## Interpretation

All local HMPs worked in all 3 focus areas during the period covered by the Outcomes Survey (FY 2003-2005). Because of the community linkages, HMPs were able to work in varied settings. Overall, schools were the most popular setting for HMP work, followed by worksites and hospitals. Overall, physical activity was the focus area targeted most by HMPs. Cumulatively, more than 4,600 policy or environmental changes were reported. Tobacco policies represent the majority of policy changes accomplished.

State efforts at program collaboration and the resulting local HMPs' efforts and progress were notable and many. The impact of the local coalitions has yet to be documented, but expecting that behaviors will change as a result of the widespread policy and environmental changes accomplished is reasonable. A new round of 3-year funding was awarded in 2007 for the HMPs.

Formation of the HMP system helped to strengthen support for using Master Settlement Agreement funds for health purposes by involving elected officials, state partners, advocates, and local coalitions. Since 2002, nearly every state legislator has had an HMP in his or her district. As a result of extensive training, technical assistance, and skill-building conferences, the HMP system has greatly improved the skills of the public health workforce at the local level. In addition to helping change social norms, HMPs have increased advocacy efforts that have resulted in positive outcomes, such as the passing of a local ordinance banning smoking in cars, which subsequently became the model for a similar state law.

The Outcome Survey methods have several limitations, respondent recall error being the primary one. Between the time HMP work was completed and data were collected in 2006, 1.5 years elapsed; retrospective reporting was therefore required. A 1-year to 2-year lapse occurred between program work and data collection for the survey administered in 2005. A second limitation is that, in some cases, local HMPs experienced staff turnover among their directors or SHCs. Therefore, staff members were encouraged to consult records, reports, data stored in the Web-based reporting system, and former staff to help them complete the survey. The state is currently implementing a new online monitoring tool so that local HMPs can track their progress more quickly as future progress is monitored. A third limitation is that no means of detecting barriers existed; only successes were counted. The new online system and the addition of a case study will allow for the documentation of contextual factors and barriers encountered. This information will help identify issues to be addressed during the second round of funding.

A final limitation is measurement error. What was counted as a policy or environmental change may differ from one respondent to another. Furthermore, it is possible that for some participants, what was counted as a change in the first year may have been counted again the second and third years. To address this issue, we provided the results of the first survey to participants as a reference so that they would not duplicate responses. In addition, the number of policies adopted may have been overestimated because policies may appear in more than one place (eg, a dress-code policy and a field-trip policy) and because policies may apply to more than one subpopulation. For example, a particular tobacco use policy at school can apply to students, staff, and visitors. If so, this policy counted as 3 policies adopted.

Despite the limitations, the data collection effort highlights notable changes made by local HMPs and suggests momentum for improving Maine's public health infrastructure. The community-based coalition approach may be applicable to other large, rural states. In Maine, training continues state-wide and regionally; an active Listserv is in place for sharing ideas and enhancing communication and networking.

## Figures and Tables

**Table 1:** Average^a^ Number of Local HMPs (N = 31) Annually Engaged in Policy and Environmental Change Efforts, by Setting and Focus Area, Healthy Maine Partnerships, FY 2003-2005

**Setting**	Focus Area		
	Tobacco	Physical Activity	Nutrition
Schools	22	30	27
Worksites	21	18	15
Hospitals	18	21	15
Municipalities	23	20	19
Colleges	12	0	0
Restaurants	0	NA	11

Abbreviations: HMPs, Healthy Maine Partnerships; FY, fiscal year; NA, not applicable.

a Average of FY 2003-2005.

**Table 2:** Most Frequently Reported Policy or Environmental Changes Adopted, by Setting, Healthy Maine Partnerships, Fiscal Years 2003-2005

**Setting**	**Tobacco**	**Physical Activity**	**Nutrition**
Schools	Enforce the "no tobacco use" policy to staff	Allow public to use outdoor walking trails on school property	Improve school menu to include healthier options
Municipalities	Prohibit all tobacco use in all municipal buildings where the public comes to conduct business	Create a new municipal walking or biking path or trail	Promote an existing farmers' market
Hospitals	Prohibit tobacco use in hospital-owned buildings Prohibit tobacco use in affiliated sites such as hospital-run clinics and doctors' offices	Establish/support an employee wellness committee	Create a policy or environmental change to include healthy options at meetings Create a policy or environmental change to include healthy options in cafeteria or snack bar
Worksites	Prohibit tobacco use in worksite buildings (including staff lounges)	Increase awareness of opportunities for physical activity	Create a policy or environmental change to include healthy options at meetings
Colleges	Communicate tobacco policy to employees, students, and the public	NA	NA
Restaurants	NA	NA	Work with restaurants to add heart-healthy options to their menus

Abbreviation: NA, not applicable.

## Appendix. Profile of Healthy Maine Partnerships (HMPs)^a^

**Table:**

**Characteristic**	FY 2003	FY 2004	FY 2005
**No. of staff positions funded, at least partially, by HMP initiative**			
Partnership directors	31	31	31
School health coordinators			
Full-time	32	28	33
Part-time	16	18	19
Youth advocacy coordinators	25	26	26
Administrative assistants	16	16	16
**No. of other positions paid for, at least partially, with HMP funds**	20	19	15
**No. of awareness-building strategies used by partnerships**			
Newspaper advertisements, paid	24	21	20
Sponsorships of other organizations or events	31	31	29
Radio advertisements	16	16	14
Local cable television advertisements	12	11	16
Newspaper articles submitted by partnership	30	30	30
Partnership Web site	18	18	19
Speaker's bureau/invited speaker at civic groups	29	30	30
Newsletters	18	20	18
E-mail notices	28	27	31
**Additional funding, $**			
Maine Office of Substance Abuse, One ME grants	1,212,867	1,310,432	1,042,912
Federal grants	184,054	598,001	3,672,637
	40,000	30,500	103,151
State agency grants (non-HMP)	611,213	196,433	109,265
Mini-grants from the state or other agency	107,584	117,652	195,232
	109,758	98,299	73,312
	69,000	202,300	34,517
	25,500	48,350	9,042
	17,489	25,800	9,777
	305,785	142,500	66,630
	49,000	96,866	21,000
Total, $	2,732,250	2,867,133	5,337,475

Abbreviation: FY, fiscal year.

a Source: Outcome Survey technical report (10).
